# Insecticide, Acaricide, Repellent and Antimicrobial Development

**DOI:** 10.3390/molecules27020386

**Published:** 2022-01-08

**Authors:** Giovanni Benelli

**Affiliations:** Department of Agriculture, Food and Environment, University of Pisa, via del Borghetto 80, 56124 Pisa, Italy; giovanni.benelli@unipi.it; Tel.: +39-0502216141

The quick spread of invasive arthropod species worldwide, sometimes boosted by global warming and urbanization [1,2,3,4], outlines again the need for effective and timely pest and vector management tools [5]. However, most of them rely on the use of synthetic insecticides and acaricides. This represents a major problem, since synthetic molecules often rely on a single mechanism of action, making resistance development quick and hard to deal with [6,7]. Similarly, fast resistance development to widely used antimicrobials has been detected in a wide number of microbial pathogens and parasites [8,9]. The massive, often inappropriate, employ of synthetic pesticides also leads to serious non-target effects on human health and the environment [10].

Further, bites from bloodsucker insects and mites can be avoided using repellents. In this scenario, discovering novel and effective products to repel mosquitoes, ticks and tabanids, just to cite some hot examples, is a challenge for public health [11,12,13,14]. Natural products represent a huge source of highly effective active ingredients to be used for repellent purposes (e.g., *Eucalyptus citriodora* and the related molecule *p*-menthane-3,8-diol) [15].

In this framework, the present Special Issue is dedicated to the development of effective and eco-friendly insecticides, acaricides, repellents and antimicrobials, including products of natural origin (e.g., plant extracts, essential oils, selected bacterial and fungal metabolites). Research efforts shedding light on the modes of action, behavioural modifications and non-target effects of the above-mentioned natural products have been welcomed. It has been recommended to the authors to include a positive control in the experiments [16], as well as detailed information on the chemical composition of the tested products [17]. Both original research and reviews have been included in the Special Issue.

Herein, contributions on the following topics have been included:

(a) Laboratory evaluation of the insecticidal, acaricidal and/or antimicrobial activity of plant essential oils [18,19].
(b) Isolation of pure constituents from plant extracts, and assessment of their insecticidal [20,21,22], acaricidal [23] and/or antimicrobial activities [24], including toxicological stability assays [25].
(c) Synthesis and characterization of novel semisynthetic insecticides, along with their in vitro evaluation on insect cells [26].
(d) Exploitation of invasive plant species as sources of effective insecticidal products [27].
(e) Evaluation of the impact of selected plant-borne compounds on the behaviour of key insect pests, with special reference to aphids [28].
(f) Development of botanical-based insecticidal formulations (including nanoformulations) characterized by an improved bioactivity and stability over time [29,30].

Finally, the Special Issue ends with two reviews. The first summarized current knowledge on the use of diatomaceous earths in crop protection, stored product, and urban pest control, presenting a number of challenges for future research [31]. The second one highlights current prospects and challenges about the use of plant-borne products as pesticides for agricultural purposes [32].

In conclusion, despite the relevant research efforts undertaken in this field for discovering new insecticides, acaricides and repellents of natural origin, the road to their large-scale use in the real world appears long and windy, complicated by costly and complex authorization requirements [33], and with limited commercialization outcomes [34]. In this scenario, I sincerely hope that the present Special Issue will be useful in inspiring future research and even extension efforts on the topic, particularly among young researchers.
